# Supplementary material for: Tissue-Specific Transcriptomes in the Secondary Cell Wall Provide an Understanding of Stem Growth Enhancement in Solidago canadensis during Invasion
Source: Biology (Basel). 2023 Oct 20;12(10):1347. doi: 10.3390/biology12101347 (PMC10604605; doi:10.3390/biology12101347)
Supplement: Supplementary file 1 [file biology-12-01347-s001.zip › Supplementary Figures.pdf]

Figure S1

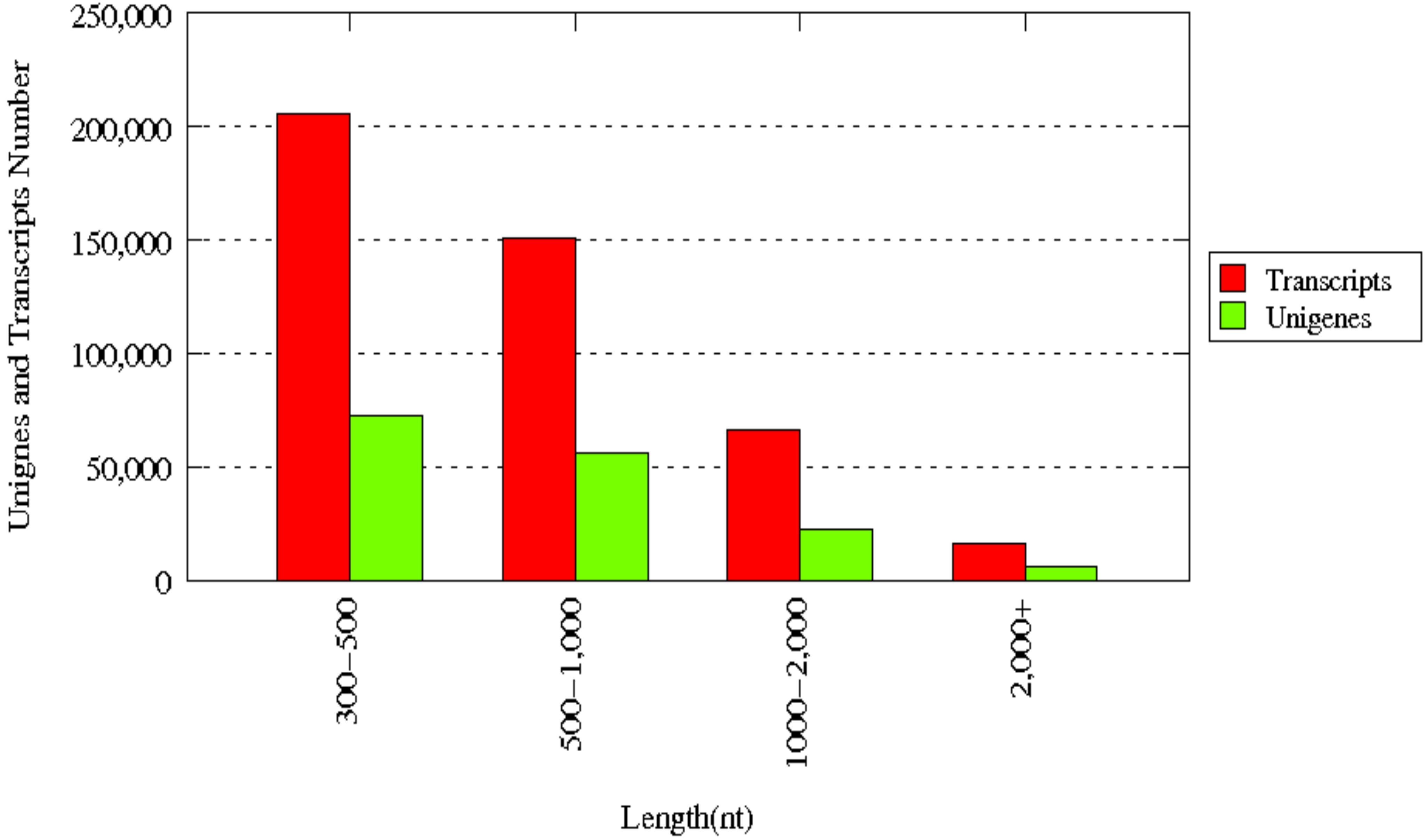

250,000
200,000
150,000
100,000
50,000
0
Unignes and Transcripts Number
Transcripts
Unigenes
300–500
500–1,000
1000–2,000
2,000+
Length(nt)

Figure S2

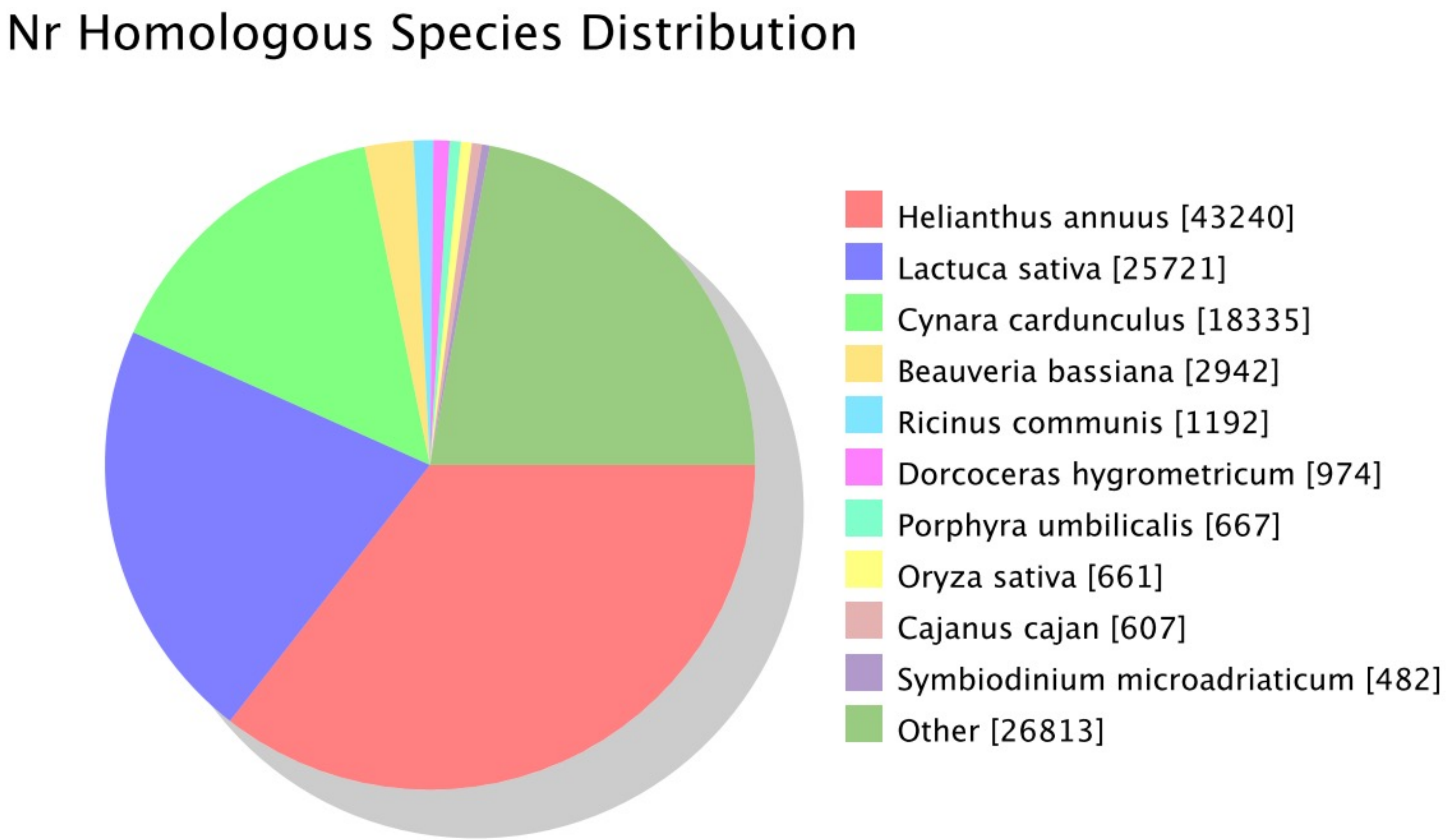
Nr Homologous Species Distribution
Helianthus annuus [43240]
Lactuca sativa [25721]
Cynara cardunculus [18335]
Beauveria bassiana [2942]
Ricinus communis [1192]
Dorcoceras hygrometricum [974]
Porphyra umbilicalis [667]
Oryza sativa [661]
Cajanus cajan [607]
Symbiodinium microadriaticum [482]
Other [26813]

Figure S3

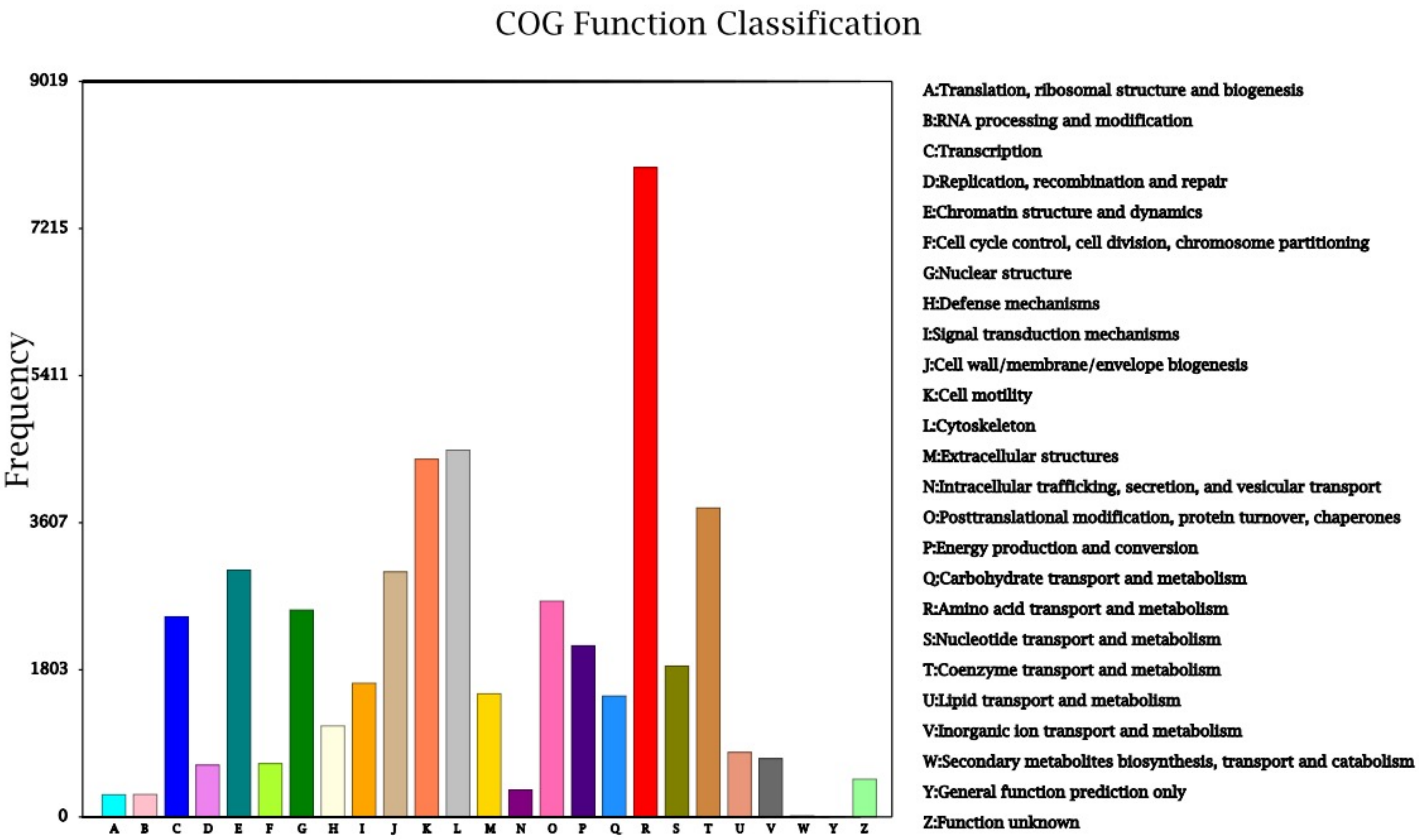
COG Function Classification
Frequency
9019
7215
5411
3607
1803
0
A B C D E F G H I J K L M N O P Q R S T U V W Y Z
A:Translation, ribosomal structure and biogenesis
B:RNA processing and modification
C:Transcription
D:Replication, recombination and repair
E:Chromatin structure and dynamics
F:Cell cycle control, cell division, chromosome partitioning
G:Nuclear structure
H:Defense mechanisms
I:Signal transduction mechanisms
J:Cell wall/membrane/envelope biogenesis
K:Cell motility
L:Cytoskeleton
M:Extracellular structures
N:Intracellular trafficking, secretion, and vesicular transport
O:Posttranslational modification, protein turnover, chaperones
P:Energy production and conversion
Q:Carbohydrate transport and metabolism
R:Amino acid transport and metabolism
S:Nucleotide transport and metabolism
T:Coenzyme transport and metabolism
U:Lipid transport and metabolism
V:Inorganic ion transport and metabolism
W:Secondary metabolites biosynthesis, transport and catabolism
Y:General function prediction only
Z:Function unknown

Figure S4

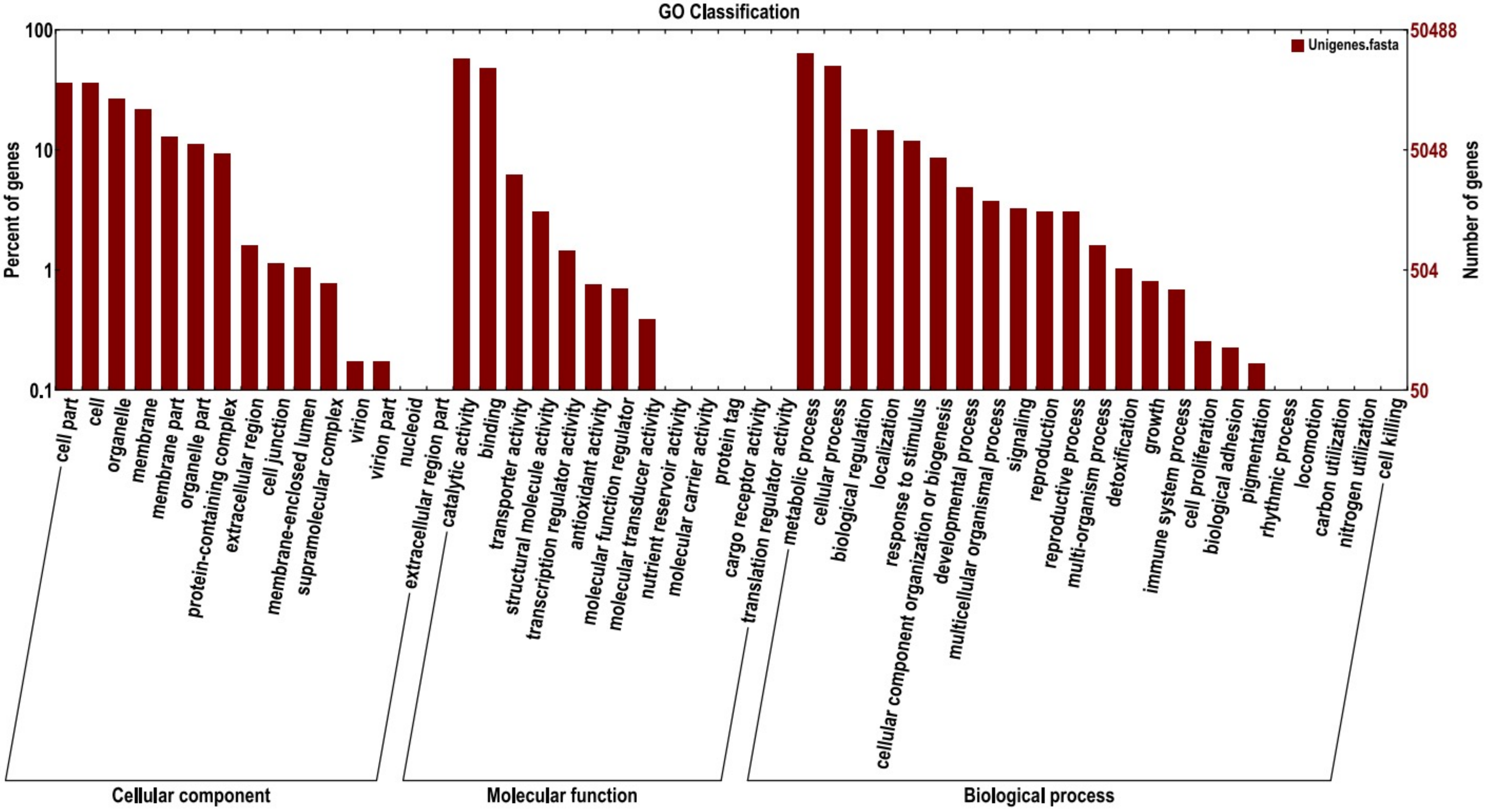
GO Classification
Unigenes.fasta
100
10
1
0.1
Percent of genes
50488
5048
504
50
Number of genes
cell part
cell
organelle
membrane
membrane part
organelle part
protein-containing complex
extracellular region
cell junction
membrane-enclosed lumen
supramolecular complex
virion
virion part
nucleoid
extracellular region part
catalytic activity
binding
transporter activity
structural molecule activity
transcription regulator activity
antioxidant activity
molecular function regulator
molecular transducer activity
nutrient reservoir activity
molecular carrier activity
protein tag
cargo receptor activity
translation regulator activity
metabolic process
cellular process
biological regulation
localization
response to stimulus
cellular component organization or biogenesis
developmental process
multicellular organismal process
signaling
reproduction
reproductive process
multi-organism process
detoxification
growth
immune system process
cell proliferation
biological adhesion
pigmentation
rhythmic process
locomotion
carbon utilization
nitrogen utilization
cell killing
Cellular component
Molecular function
Biological process

Figure S5

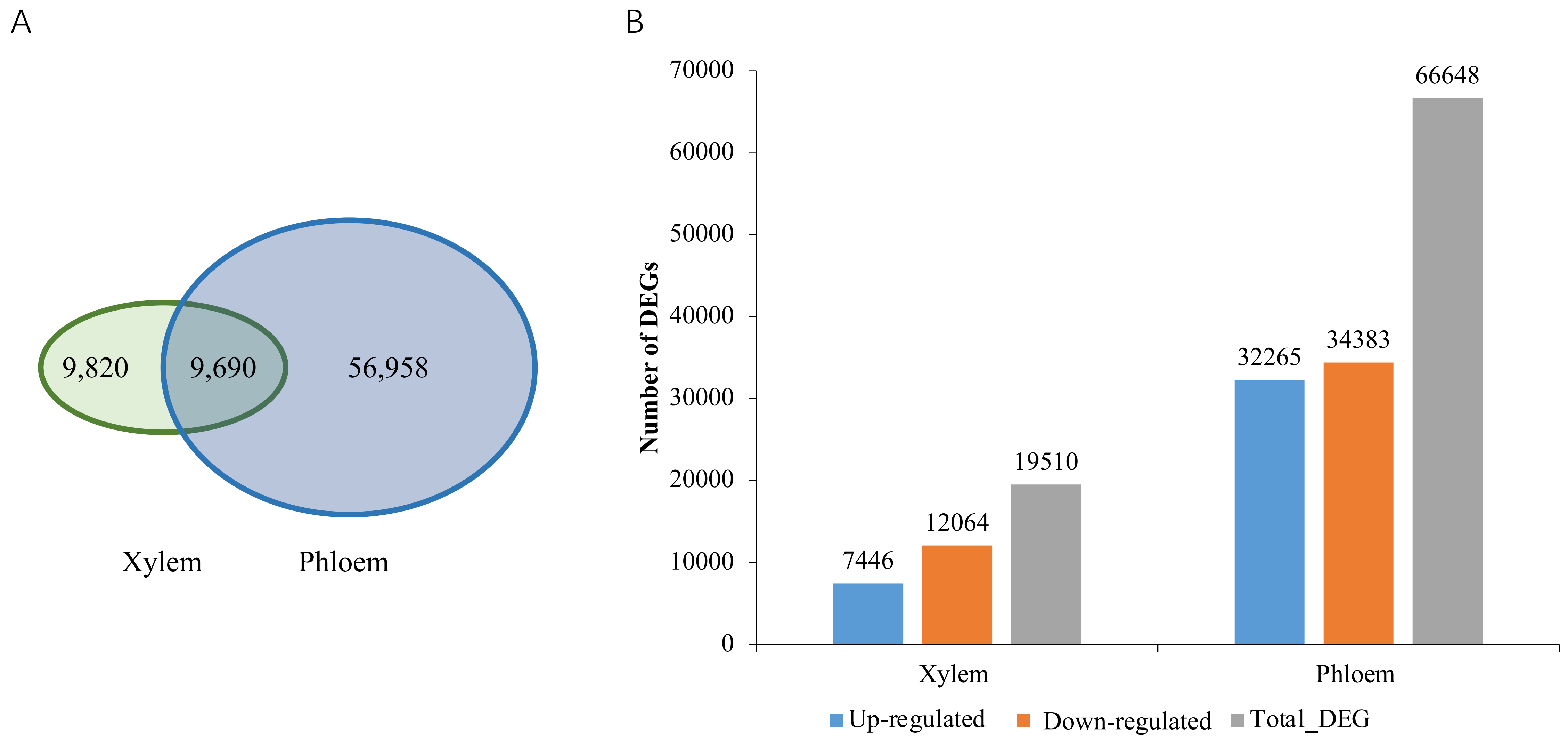
A
9,820
9,690
56,958
Xylem
Phloem
B
Number of DEGs
70000
60000
50000
40000
30000
20000
10000
0
7446
12064
19510
32265
34383
66648
Xylem
Phloem
Up-regulated
Down-regulated
Total_DEG

Figure S6

Synthase type	CesA-related group	Gene ID	Tissue: Phl US01	Phl CN25	Phl log2(CN25/US01)	Xyl	Xyl CN25	Xyl log2(CN25/US01)
PCW CesA	CesA1	Unigene158698	16	201	3.6	0	0	0.0
		Unigene042732	0	9	3.5	0	0	0.0
		Unigene035899	139	1290	3.2	1166	431	-1.4
		Unigene044965	27	235	3.1	178	71	-1.3
		Unigene192572	24	129	2.4	0	0	0.0
		Unigene060826	12	66	2.4	0	0	0.0
		Unigene023374	24	99	2.1	0	0	0.0
		Unigene128082	22	88	2.0	80	33	-1.3
		Unigene002836	6	20	1.7	0	0	0.0
		Unigene093804	816	2195	1.4	2019	771	-1.4
		Unigene118578	0	0	0.0	7	0	-3.0
		Unigene050986	0	0	0.0	2167	686	-1.7
		Unigene191810	65	15	-2.1	10	2	-2.0
		Unigene197427	9	0	-4.6	0	0	0.0
	CesA2	Unigene052439	0	4	3.0	0	0	0.0
		Unigene076361	62	291	2.2	0	0	0.0
		Unigene195770	3	15	2.1	15	1	-2.9
		Unigene200563	208	21	-3.3	0	0	0.0
		Unigene170658	142	2	-5.7	0	0	0.0
	CesA3	Unigene139315	0	4	3.2	14	1	-3.0
		Unigene079925	3528	7383	1.1	0	0	0.0
	CesA6	Unigene163623	536	2343	2.1	1799	891	-1.0
		Unigene013975	0	0	0.0	517	197	-1.4
SCW CesA	CesA4	Unigene081771	7	90	3.6	1557	3909	1.3
		Unigene105545	9	89	3.2	0	0	0.0
		Unigene017813	2	23	3.1	7	32	2.1
		Unigene165767	10	60	2.6	1132	2606	1.2
		Unigene163327	14	1	-3.0	31	74	1.2
		Unigene038015	115	8	-3.9	0	0	0.0
		Unigene106927	40	1	-4.8	0	0	0.0
	CesA7	Unigene061691	0	35	6.3	444	1280	1.5
		Unigene062101	0	18	5.4	327	1181	1.9
		Unigene113186	5	163	5.0	0	0	0.0
		Unigene087618	9	155	4.0	0	0	0.0
		Unigene040529	3	12	1.9	0	0	0.0
		Unigene185079	4	0	-3.1	0	0	0.0
	CesA8	Unigene013956	0	8	4.1	171	474	1.5
		Unigene105993	0	6	3.7	136	351	1.4
		Unigene034707	7	67	3.1	0	0	0.0
		Unigene113775	14	102	2.8	0	0	0.0
		Unigene202406	105	0	-7.9	0	0	0.0
	CesA9	Unigene014207	0	0	0.0	33	77	1.2
		Unigene063809	0	0	0.0	3440	1389	-1.3

Synthase type	CesA-related group	Gene ID	Tissue: Phl US01	Phl CN25	Phl log2(CN25/US01)	Xyl US01	Xyl CN25	Xyl log2(CN25/US01)
CSLB	CSLB4	Unigene035504	0	0	0.0	5	0	-3.2
CSLD	CSLD1	Unigene040624	0	0	0.0	18	43	1.2
	CSLD3	Unigene033687	2	9	2.0	38	6	-2.6
		Unigene046830	8	22	1.4	78	17	-2.1
		Unigene182415	11	35	1.6	135	27	-2.3
		Unigene058356	0	0	0.0	1848	285	-2.7
		Unigene144078	0	0	0.0	1892	297	-2.7
	CSLD4	Unigene079242	0	0	0.0	18	52	1.5
		Unigene126423	0	0	0.0	50	103	1.0
	CSLD5	Unigene165411	22	47	1.1	0	0	0.0
		Unigene045301	23	64	1.4	0	0	0.0
CSLE	CSLE1	Unigene059776	7	38	2.4	0	0	0.0
		Unigene173941	58	1	-5.0	0	0	0.0
		Unigene088118	0	0	0.0	133	50	-1.4
	CSLE2	Unigene037260	77	164	1.1	0	0	0.0
	CSLE6	Unigene140739	118	4	-4.7	0	0	0.0
CSLG	CSLG1	Unigene125327	14	0	-4.5	0	0	0.0
	CSLG2	Unigene173005	1755	288	-2.6	0	0	0.0
		Unigene162276	241	24	-3.3	0	0	0.0
		Unigene193334	0	0	0.0	321	116	-1.5
		Unigene160427	0	0	0.0	78	27	-1.5
		Unigene012160	0	0	0.0	629	219	-1.5
		Unigene017926	0	0	0.0	451	138	-1.7
	CSLG3	Unigene041967	39	116	1.6	0	0	0.0
		Unigene023737	27	57	1.1	0	0	0.0
		Unigene070487	445	145	-1.6	0	0	0.0
		Unigene182179	50	12	-2.0	0	0	0.0
CSLH	CSLH1	Unigene109281	40	4	-3.2	9	0	-4.0
	CSLH2	Unigene134350	32	1	-5.2	0	0	0.0
		Unigene177899	49	1	-5.3	0	0	0.0
		Unigene014506	239	4	-5.8	0	0	0.0

0 50 100 150 200 300 400

Colour scale indicating RNA expression level as FPKM values

-6 -5 -4 -3 -2 -1 0 1 2 3 4 5 6

Colour scale indicating expression ration Log2(fold change CN25/US01)
